# Long-Distance Delivery of Bacterial Virulence Factors by *Pseudomonas aeruginosa* Outer Membrane Vesicles

**DOI:** 10.1371/journal.ppat.1000382

**Published:** 2009-04-10

**Authors:** Jennifer M. Bomberger, Daniel P. MacEachran, Bonita A. Coutermarsh, Siying Ye, George A. O'Toole, Bruce A. Stanton

**Affiliations:** 1 Department of Physiology, Dartmouth Medical School, Hanover, New Hampshire, United States of America; 2 Department of Microbiology and Immunology, Dartmouth Medical School, Hanover, New Hampshire, United States of America; Massachusetts General Hospital, United States of America

## Abstract

Bacteria use a variety of secreted virulence factors to manipulate host cells, thereby causing significant morbidity and mortality. We report a mechanism for the long-distance delivery of multiple bacterial virulence factors, simultaneously and directly into the host cell cytoplasm, thus obviating the need for direct interaction of the pathogen with the host cell to cause cytotoxicity. We show that outer membrane–derived vesicles (OMV) secreted by the opportunistic human pathogen *Pseudomonas aeruginosa* deliver multiple virulence factors, including β-lactamase, alkaline phosphatase, hemolytic phospholipase C, and Cif, directly into the host cytoplasm via fusion of OMV with lipid rafts in the host plasma membrane. These virulence factors enter the cytoplasm of the host cell via N-WASP–mediated actin trafficking, where they rapidly distribute to specific subcellular locations to affect host cell biology. We propose that secreted virulence factors are not released individually as naked proteins into the surrounding milieu where they may randomly contact the surface of the host cell, but instead bacterial derived OMV deliver multiple virulence factors simultaneously and directly into the host cell cytoplasm in a coordinated manner.

## Author Summary

Gram-negative pathogens are responsible for 2 million annual hospital-acquired infections, adding tremendously to U.S. healthcare costs. *Pseudomonas aeruginosa*, an opportunistic human pathogen, is commonly associated with nosocomial infections, particularly ventilator-associated infections and pseudomonal pneumonia in immunocompromised patients with cystic fibrosis, chronic obstructive pulmonary disease, ventilator-associated pneumonia, community-acquired pneumonia, and bronchiectasis. We have identified the mechanism for a secretion system that Gram-negative bacteria use to strategically deliver toxins to the host to promote bacterial virulence and host colonization, a pathway that we hope to target to develop new therapies to treat *P. aeruginosa* infections. Our findings have significant implications for the study of Gram-negative bacterial pathogenesis. We propose that secreted virulence factors are not released individually as naked proteins into the surrounding milieu where they may randomly contact the surface of the host cell, but instead bacterial-derived outer membrane vesicles (OMV) deliver multiple virulence factors simultaneously and directly into the host cell cytoplasm in a coordinated manner. This long-distance bacterial communication to the host via OMV is reminiscent of the delivery of signaling proteins and miRNA between eukaryotic cells via exosomes, and may represent a general protein secretion strategy used by both pathogen and host.

## Introduction

Nosocomial infections contribute $4.5 billion to annual healthcare costs in this country alone, with an estimated 2 million nosocomial infections occurring in the US annually, resulting in 99,000 deaths [1]. Many of these nosocomial infections are caused by Gram-negative pathogens, and interaction of these pathogens with the host is often mediated by secreted virulence factors. Bacteria have evolved mechanisms for the secretion of virulence factors into the host cell to alter host cell biology and enable bacterial colonization, and these mechanisms typically require that bacteria be in intimate contact with the host. For example, the Type III secretion system (T3SS) and Type IV secretion system (T4SS) deliver proteins directly into the host cytoplasm from an extracellular bacterial pathogen's cytoplasm [2] utilizing transport machines that act as macromolecular syringes [3]. Delivery of extracellular bacteria or bacterial products can also occur via endocytosis initially into the lumen of the host endocytic compartment, then movement to the host cytoplasm via lysis of the endocytic compartment or delivery of the proteins across the endocytic membrane via the Type III Secretion System (T3SS) [3].

For several decades, work by Beveridge's group has characterized bacterial-derived outer membrane vesicles (OMV) to be a novel secretion mechanism employed by bacteria to deliver various bacterial proteins and lipids into host cells, eliminating the need for bacterial contact with the host cell [4]–[7]. OMV are 50–200 nm proteoliposomes constitutively released from pathogenic and non-pathogenic species of Gram-negative bacteria [8],[9]. Biochemical and proteomic analyses have revealed that OMV are comprised of lipopolysaccharide, phospholipids, outer membrane proteins, and soluble periplasmic proteins [8],[9]. Many virulence factors that are periplasmic proteins are enriched in OMV, for example, *Escherichia coli* cytolysin A (ClyA), enterotoxigenic *E. coli* heat labile enterotoxin (LT), and *Actinobacillus actinomycetemcomitans* leukotoxin [10]–[12]. Beveridge's group and others have reported that some secreted virulence factors from *P. aeruginosa*, including β-lactamase, hemolytic phospholipase C, alkaline phosphatase, pro-elastase, hemolysin, and quorum sensing molecules, like *N*-(3-oxo-dodecanoyl) homoserine lactone and 2-heptyl-3-hydroxy-4-quinolone (PQS) [6],[7],[13],[14], are also associated with *P. aeruginosa* OMV [8],[9]. Whether these secreted virulence factors packaged in OMV are eventually delivered to the host and the mechanism by which this occurs is currently unknown. A recent study suggested that *E. coli* OMV fuse with lipid rafts in the host colonic epithelial cell, but the delivery and intracellular trafficking of the OMV cargo was not characterized [15]. Thus, we investigated the possibility that OMV deliver multiple secreted virulence factors into the host cell through a lipid raft-mediated pathway, eliminating the need for intimate contact of the pathogen with the host.

## Results

### Outer membrane vesicles deliver toxins to airway epithelial cells

**Figure 1:**
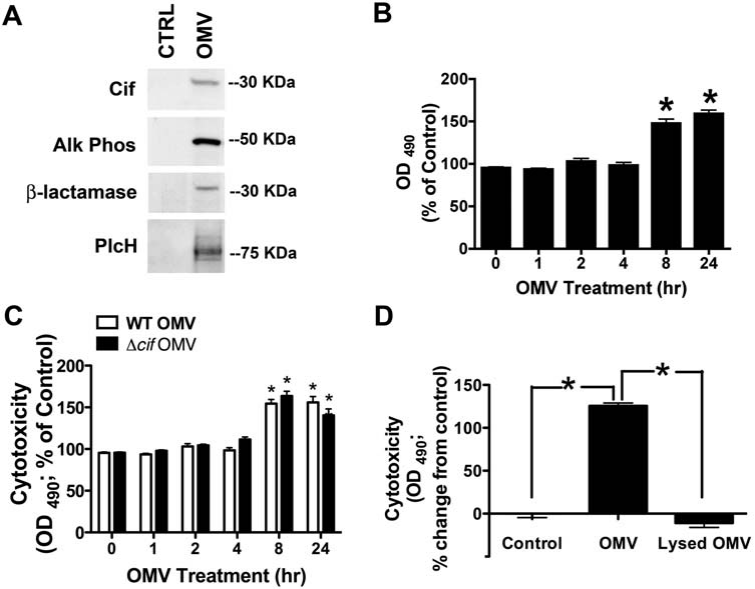
OMV deliver multiple toxins to induce cytotoxicity in airway epithelial cells. (A) Purified *P. aeruginosa* OMV deliver multiple virulence factors (Cif, alkaline phosphatase, β-lactamase, and PlcH) into the cytoplasm of airway epithelial cells. Western blot analysis of cell lysates of airway epithelial cells treated with purified OMV. CTRL, no OMV added. Δ*cif* OMV applied to airway epithelial cells, cells lysed, and probed by Western blot analysis for Cif did not detect Cif in airway cell lysates (data not shown). (B) OMV elicit time-dependent cytotoxicity in host airway cells as determined by the CellTiter 96 AQ_ueous_ One cytotoxicity assay. (C) OMV elicit time-dependent cytotoxicity in host airway cells that is not dependent on the presence of Cif, as determined by the CellTiter 96 AQ_ueous_ One cytotoxicity assay. Δ*cif* OMV, OMV purified from a strain of *P. aeruginosa* lacking the *cif* gene. Black bars, wild-type OMV (WT OMV); white bars, Δ*cif* OMV. (D) Airway epithelial cells treated with lysed OMV components demonstrated a reduction in cellular cytotoxicity as determined by the CellTiter 96 AQ_ueous_ One cytotoxicity assay. Data are presented as mean+/−SEM. n = 3, * p<0.05, OMV versus Control, intact OMV versus lysed OMV.

Based on reports that multiple virulence factors are packaged in OMV, we hypothesized that these virulence factors could be simultaneously delivered in a coordinated manner in OMV to the host cell by the microbe. We tracked four *P. aeruginosa* secreted factors, including alkaline phosphatase, β-lactamase, hemolytic phospholipase C, and Cif, previously reported to be packaged in OMV [6],[7],[13],[14],[16]. We chose these secreted virulence factors because they play important roles in host colonization, for example alkaline phosphatase promotes biofilm formation [17],[18], β-lactamase degrades host antimicrobial peptides, hemolytic phospholipase C is cytotoxic and promotes *P. aeruginosa* virulence [19], and Cif is a recently characterized toxin that inhibits CFTR-mediated chloride secretion in the airways [16] and thereby likely reduces mucociliary clearance. To purify OMV from bacterial products not packaged in OMV, like pilus, that may also elicit a host response, we modified a published protocol [14] utilizing high-speed differential centrifugation and density gradient fractionation to isolate OMV from an overnight *P. aeruginosa* culture supernatant. The Cif protein, as well as a protein present in the membrane of OMV, Omp85 [20], were identified in purified bacterial-derived OMV (Figure S1). When airway cells were treated with isolated and purified OMV for ten minutes all four OMV proteins examined were detected in host airway epithelial cell lysate (Figure 1A). By contrast, these virulence factors were not detected in lysates of control cells treated with vehicle (Figure 1A). Therefore, OMV deliver multiple virulence factors to host airway epithelial cells in the absence of bacteria, thus providing a mechanism for bacteria to alter host cell physiology without the need for intimate contact with the host.

To explore the significance of OMV in the delivery of virulence factors into the host cytoplasm, we examined the cytotoxic effect of *P. aeruginosa* OMV on host airway cells using the CellTiter 96 AQ_ueous_ One cytotoxicity assay. OMV were cytotoxic after a delay of 8 hours (Figure 1B), although virulence factors could be detected in the cytoplasm of host cells after 10 minutes (Figure 1A). The time-dependent increase in cytotoxicity induced by OMV was not dependent on Cif expression in OMV, given that Δ*cif* OMV did not produce a statistically significant difference in cytotoxicity compared to wild-type OMV (Figure 1C). To determine if intact OMV are required for cytotoxicity, purified OMV were lysed with 0.1 M EDTA and the lysate was applied to airway epithelial cells for 8 h (Figure S2, Figure 1D). This method was previously employed by Horstman *et al.* to effectively lyse *E. coli* OMV [21]. The lysed OMV did not have a cytotoxic effect on airway epithelial cells, demonstrating that cytotoxicity is mediated by virulence factors delivered into the host cell cytoplasm by bacterial-derived OMV (Figure 1D).

In the next series of experiments we began to examine the mechanism whereby OMV deliver virulence factors into the cytoplasm of the host airway epithelial cell. Previously we reported that purified, recombinant Cif, a virulence factor secreted in OMV by *P. aeruginosa*, is necessary and sufficient to reduce apical membrane expression of CFTR and P-glycoprotein (Pgp) in human airway epithelial cells [16],[22], thus reducing mucociliary clearance and xenobiotic resistance of the host cells, respectively. In the current study we use Cif as a model protein to investigate how OMV deliver virulence factors into the cytoplasm of human airway epithelial cells. First, experiments were conducted to confirm our previous observation that Cif is secreted in purified OMV and second, to determine if Cif is an intravesicular component of OMV. Cif was detected in OMV derived from *P. aeruginosa* expressing the *cif* gene, but not in OMV derived from *P. aeruginosa* in which the *cif* gene was deleted (Figure S3). The inability of Proteinase K (0.1 µg/ml), which does not enter the lumen of OMV, to degrade OMV-associated Cif indicates that this virulence factor is an intravesicular component of OMV (Figure S3). Thus, these studies demonstrate that Cif maintains an intravesicular localization in purified OMV.

**Figure 2:**
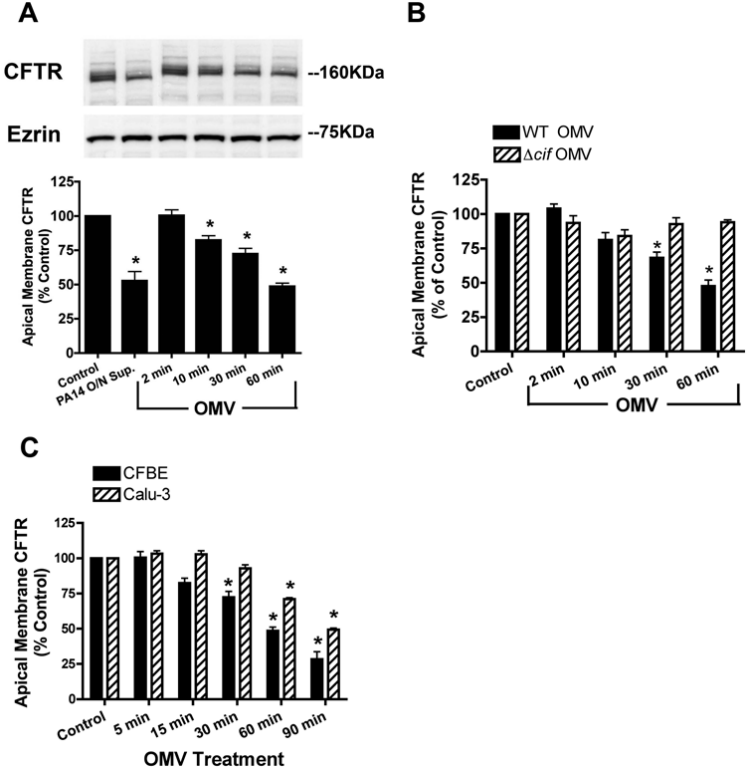
OMV deliver functional Cif virulence factor to airway epithelial cells, across a mucus layer. (A) Purified OMV applied to the apical surface of airway epithelial cells reduce CFTR in the plasma membrane in a time-dependent manner, compared to the buffer control (Control), as measured by Western blot analysis. Overnight supernatant (PA14 O/N Sup), previously reported to decrease apical membrane CFTR, serves as a positive control [16]. (B) OMV purified from a *P. aeruginosa* Δ*cif* mutant strain did not reduce apical membrane expression of CFTR, as measured by Western blot analysis. Black bar, wild-type OMV; striped bar, Δ*cif* OMV. (C) Cif-containing OMV decrease apical membrane CFTR in airway epithelial cells (CFBE) and mucous-producing airway epithelial cells (Calu-3), as measured by Western blot analysis. Black bar, CFBE cells; striped bar, Calu-3 cells. Data are presented as mean+/−SEM. n = 3, * p<0.05 versus Control.

Next, studies were conducted to determine if the Cif virulence factor packaged in OMV was functional when delivered to airway epithelial cells. Cif function was measured by examining the ability of Cif to reduce apical plasma membrane CFTR abundance in airway epithelial cells. Purified OMV containing Cif reduced apical plasma membrane CFTR in a time-dependent manner (Figure 2A), whereas purified OMV from *P. aeruginosa* deleted for the *cif* gene had no effect on CFTR membrane expression (Figure 2B). Taken together these studies confirm and extend our previous observations that OMV-packaged Cif reduces plasma membrane CFTR.

If OMV modulate host physiology of lung cells without direct bacteria-host contact, we would predict that OMV secreted by bacteria should overcome barriers such as the mucus overlying human airway cells [23]. OMV containing Cif reduced CFTR in the apical plasma membrane of airway epithelial cells that have a thick layer of mucus on the apical surface (Calu-3 cells, Figure 2C). A delay in the Cif-mediated reduction of apical membrane CFTR abundance was observed in Calu-3 cells, compared to airway cells that lack a overlying mucus layer, suggesting the mucus only delays OMV from diffusing to host airway epithelial cells. Thus, OMV allow the long distance delivery of secreted bacterial factors to the host cell in the absence of direct bacteria-host contact.

### Host cell detergent-resistant membranes (aka, lipid rafts) are required for OMV fusion and toxin delivery

**Figure 3:**
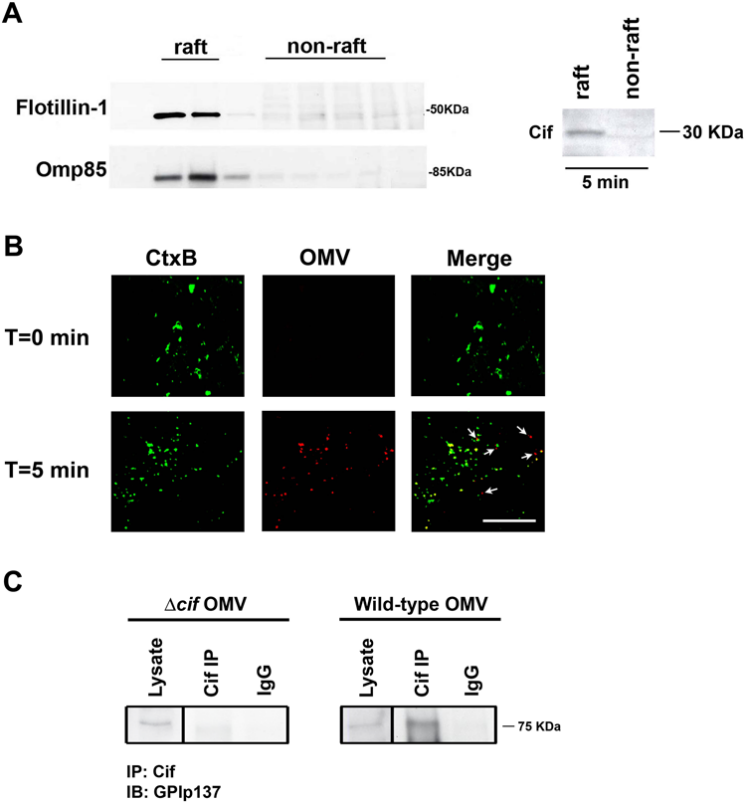
Cif-containing OMV fuse with the epithelial cell lipid raft microdomains. (A) Cif virulence factor and Omp85 localize to the lipid raft fraction. Omp85, a documented OMV protein, and Cif virulence factor co-fractionate with the lipid raft marker, flotillin-1, when raft and non-raft fractions are isolated from airway epithelial cells treated with OMV for 5 min, as measured by Western blot analysis. Right blot represents raft and non-raft fractions from the left blot, which are combined and the proteins precipitated to concentrate Cif signal to allow antibody detection. (B) Rhodamine R18-labeled OMV, which only fluoresce red upon fusion with the host plasma membrane, co-localize with the FITC-conjugated lipid raft marker cholera toxin B subunit (CtxB). All OMV co-localize with lipid rafts with the exception of five OMV that are indicated with small white arrows (see lower right panel). Scale bar equals 10 µm. (C) Cif virulence factor interacts with GPIp137 in the lipid raft fraction. Immunoprecipitation (IP) experiments demonstrate that the GPI-anchored protein p137 (GPIp137), a lipid raft marker, interacts with Cif in the lipid raft fractions of airway epithelial cells treated with wild-type OMV for 5 min. Serving as a negative control, *cif* mutant OMV (Δ*cif* OMV)–treated airway epithelial cells cannot immunoprecipitate Cif and therefore do not demonstrate immunoprecipitation of GPIp137. 5% of total lysate run in Lysate lane. IB, immunoblot. Experiments repeated three times; representative blots/images are shown.

We next explored the mechanism whereby OMV deliver bacterial proteins into the host cell using the Cif virulence factor as a model. Based on a recent study showing that Filipin III disrupts *E. coli* OMV association with host cells [15], we hypothesized that OMV deliver secreted bacterial proteins to host cells by fusing with lipid raft microdomains. Five minutes after addition of OMV to epithelial cells, Cif, as well as a protein documented to be associated with OMV, Omp85 [20], were detected in membrane lipid raft fractions (Figure 3A). The lipid raft (i.e. detergent insoluble membranes) fractions were separated with density gradient fractionation and characterized by labeling with the flotillin-1 antibody. The fusion of OMV with membrane rafts was observed visually by confocal microscopy using cholera toxin B subunit (labeled with FITC), a documented lipid raft marker [24], which co-localized with rhodamine-R18 labeled OMV five minutes after OMV were added to the apical side of airway cells (Figure 3B). The rhodamine-R18 dye is quenched when loaded in bilayer membranes at a high concentration and is subsequently dequenched, fluorescing in the red channel upon membrane fusion, which allows dilution of the probe and fluorescence detection. Pearson's correlation and Mander's overlap coefficients demonstrated a high degree of co-localization of cholera toxin B subunit and OMV (0.771+/−0.018 and 0.952+/−0.012 versus control levels of 0.153+/−0.026 and 0.259+/−0.026, respectively, p<0.0001). In further support that Cif-containing OMV fuse with lipid rafts, Cif co-immunoprecipitated with the glycosylphosphatidylinisotol-anchored protein p137 [25], a documented lipid raft-associated protein, from the lipid raft fraction of airway cells that had been treated with OMV for five minutes (Figure 3C).

**Figure 4:**
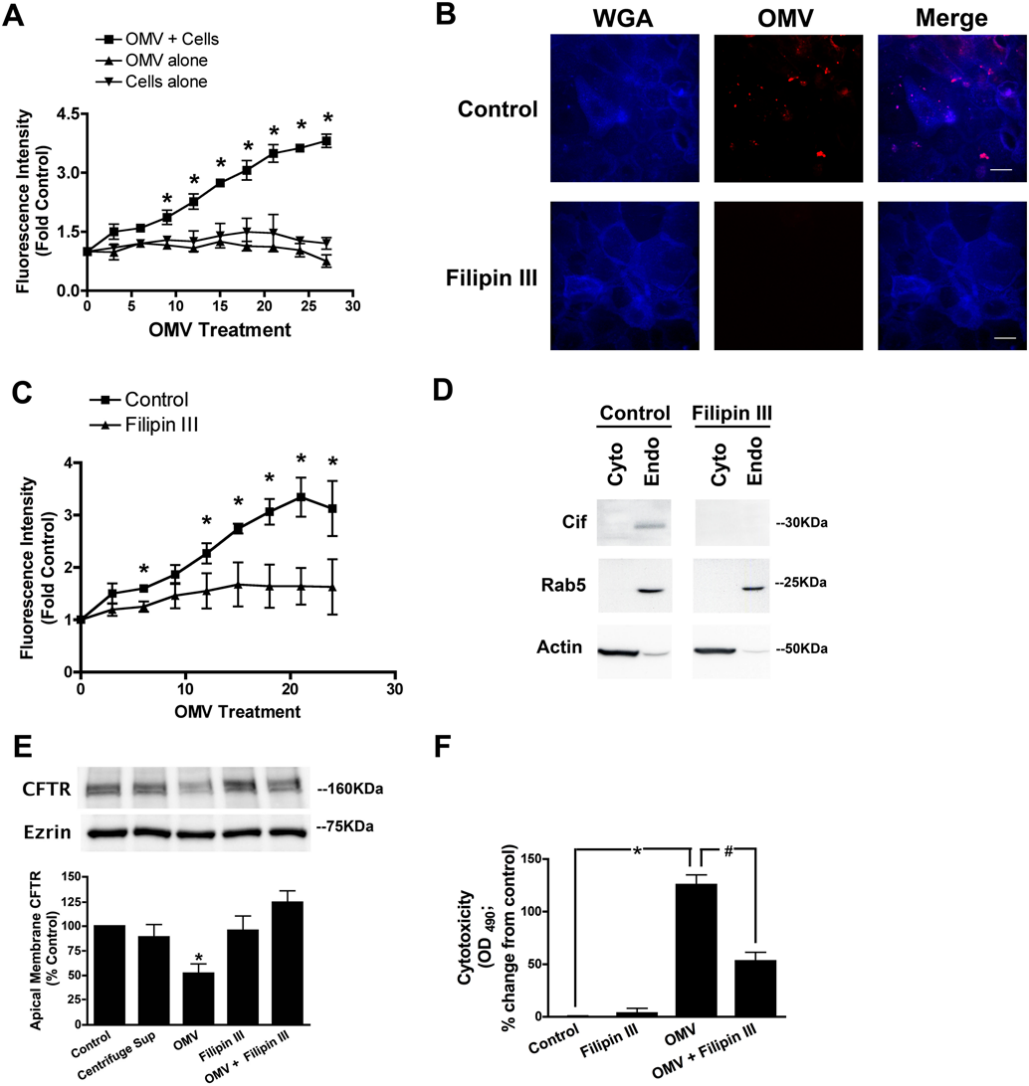
Disruption of lipid rafts blocks virulence factor delivery and function in airway epithelial cells. (A) Rhodamine-R18 labeled OMV were applied to the apical side of polarized airway epithelial cells, and fluorescence was measured over time as readout for OMV fusion. Rhodamine-R18 signal is quenched at high concentration in OMV, but fluorescence increases as the rhodamine probe is diluted by fusion of the OMV with the host plasma membrane. OMV and cells alone serve as negative controls where the fluorescence does not change over time (background fluorescence). Black square, OMV+cells; black triangle, OMV alone; black inverted triangle, cells alone. Data are presented as mean fluorescence intensity. (B,C) Visualization and quantitative assay demonstrating that disruption of lipid rafts with the cholesterol-sequestering reagent, filipin III complex (10 µg/ml), inhibits OMV fusion with airway epithelial cells. Rhodamine-R18 labeled OMV were applied to the apical side of polarized airway epithelial cells in the presence or absence of filipin III, and fluorescence was measured over time. Wheat germ agglutin (WGA-blue) is a marker of host epithelial cell membranes. Scale bars equal 10 µm. Black square, control; black triangle, filipin III. (D) Filipin III prevents delivery of Cif to host cell endosomes, as measured by cytoplasmic and endosomal fractionation and Western blot analysis. Rab5 GTPase and actin are endosomal and cytoplasmic markers, respectively. Cyto, cytoplasm; Endo, endosome. (E) Disruption of lipid rafts blocks Cif-mediated reduction in apical membrane CFTR expression, compared to buffer control (Control), as measured by Western blot analysis. “Centrifuge Sup” refers to the overnight culture supernatant of *P. aeruginosa* PA14 depleted of OMV, which serves as a negative control in this experiment. (F) Filipin III complex disruption of host cell lipid raft microdomains reduces the cytotoxic effect of OMV on host airway cells with 8 h OMV treatment, as measured with the CellTiter 96 AQ_ueous_ One cytotoxicity assay. Data are presented as mean+/−SEM. n = 3, * p<0.05, OMV versus Control; # p<0.05, OMV versus OMV+Filipin III.

To determine if host cell lipid raft microdomains are required for OMV fusion, the cholesterol-sequestering agent Filipin III complex was used to disrupt lipid raft domains and OMV fusion was assessed. The rhodamine-R18 dye was utilized to allow visualization and quantitation of OMV fusion with host cells. Rhodamine-R18 only fluoresces upon OMV fusion to host cells, thus an increase in fluorescence is interpreted as an increase in OMV fusion. Rhodamine-R18 labeled OMV applied to the apical membrane of airway epithelial cells produced a time-dependent increase in fluorescence (Figure 4A). In contrast, the fluorescence did not increase above background levels in samples containing only airway epithelial cells or only rhodamine-R18 labeled-OMV (Figure 4A). Filipin III eliminated the fusion of OMV with epithelial cells, indicated by a lack of fluorescence detected when compared to control epithelial cells (Figure 4B). Microscopy studies were confirmed by the quantitative, fluorescence-based assay, described in Figure 4A, which also demonstrated that OMV fusion to the host cell was blocked with Filipin III pretreatment of the host cells (Figure 4C).

We next tracked the Cif virulence factor biochemically to determine if lipid raft microdomains are required for virulence factor delivery and function in host cells. Five minutes after OMV are exposed to host airway epithelial cells, the Cif virulence factor is detected by Western blot analysis in the endosomal sub-fraction of the host cell lysate, but not in the cytoplasmic fraction (Figure 4D). The Filipin III complex prevented the appearance of Cif in the endosomal fraction of host cells (Figure 4D) and blocked the ability of Cif to reduce apical membrane CFTR (Figure 4E), demonstrating a requirement for the host lipid raft machinery for Cif delivery and function in host cells. Furthermore, disruption of lipid raft microdomains with Filipin III, and thus blocking OMV fusion with airway epithelial cells, reduced the cytotoxicity induced in the airway epithelial cells with 8 h of OMV treatment (Figure 4F). Thus, lipid raft microdomains are required for OMV-mediated delivery and function of secreted virulence factors in host cells.

### Actin cytoskeleton is required for OMV fusion, toxin entry, and function

**Figure 5:**
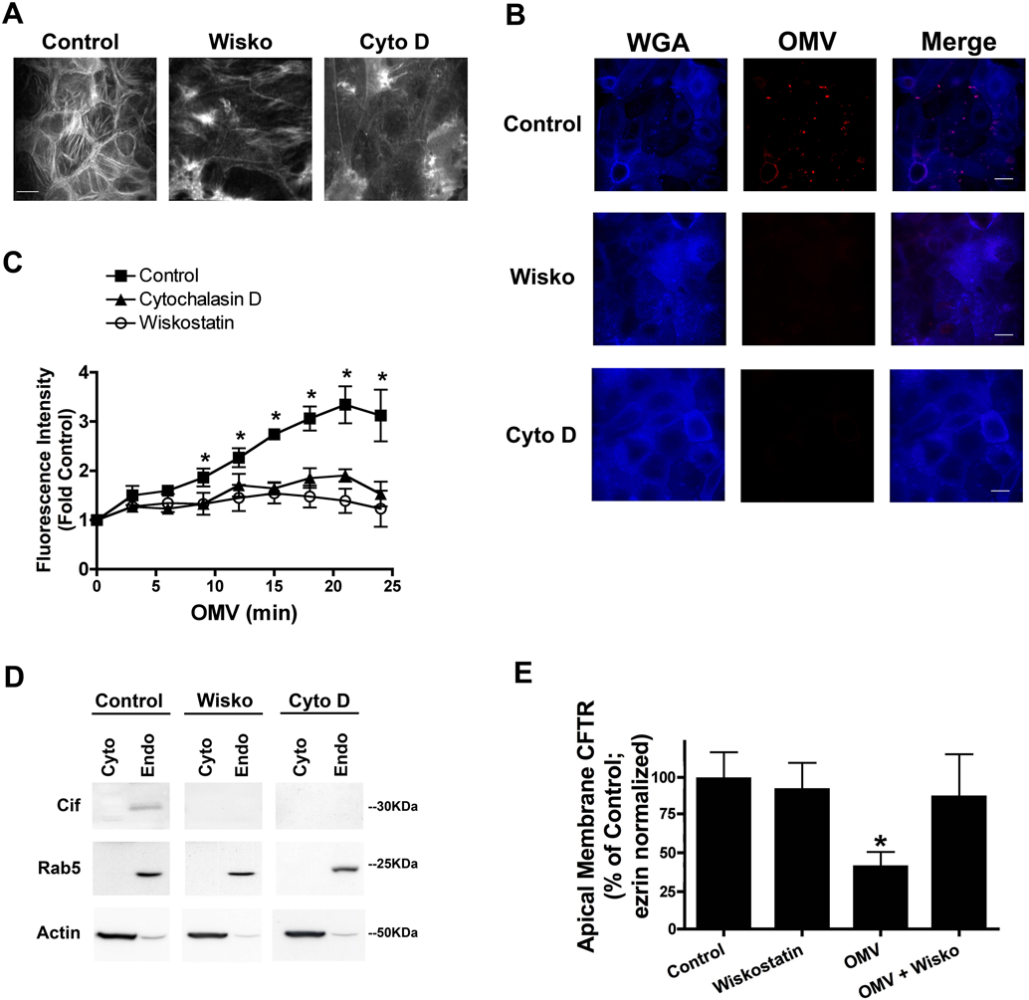
Intact N-WASP–induced actin cytoskeleton is required for OMV fusion and virulence factor delivery. (A) Wiskostatin and cytochalasin D disrupt actin cytoskeleton in airway cells. Alexa 647–conjugated phalloidin was used to label actin to monitor disruption of the actin cytoskeleton by two actin-disrupting agents, wiskostatin (Wisko, 10 µM for 30 min) and cytochalasin D (Cyto D, 2 µM for 45 min), by immunofluorescence microscopy. Scale bar equal to 20 µm. (B,C) Visualization and quantitative assay demonstrating that disruption of the actin cytoskeleton with either of the two actin-disrupting agents, wiskostatin (Wisko, 10 µM) or cytochalasin D (Cyto D, 2 µM), inhibits OMV fusion with airway epithelial cells. Rhodamine-R18 labeled OMV were applied to the apical side of polarized airway epithelial cells in the presence or absence of wiskostatin or cytochalasin D, and fluorescence was measured over time. Scale bar equals 10 µm (B). Representative images of three experiments revealing that OMV do not fuse with cells in which actin has been disrupted. WGA, wheat germ agglutin (blue) to label the cell surface. Black square, control; black triangle, Cytochalasin D; open circle, Wiskostatin. * p<0.05 (Control versus Cytochalasin D and Wiskostatin). (D) Wiskostatin or cytochalasin D treatment of airway epithelial cells prevents delivery of Cif virulence factor to endosomal fraction, as measured by cytoplasm and endosome fractionation and Western blot analysis. Rab5 GTPase and actin are endosomal and cytoplasmic markers, respectively. Cyto, cytoplasm; Endo, endosome. (E) Disruption of the actin cytoskeleton with wiskostatin blocks the Cif virulence factor–mediated reduction in CFTR apical membrane expression in airway epithelial cells, as measured by Western blot analysis. Data are presented as mean+/−SEM. n = 3, * p<0.05 versus Control.

The actin cytoskeleton, in particular the neuronal WASP (N-WASP)–initiated actin assembly, is critical to the internalization of select lipid raft-associated cargo [26],[27]. Based on these previous studies, we investigated the role of the actin cytoskeleton, in general, and N-WASP-mediated cytoskeletal rearrangements specifically, in OMV fusion to the plasma membrane of the airway epithelial cell. Both cytochalasin D (an actin monomer-sequestering agent) and wiskostatin (an inhibitor of neuronal WASP (N-WASP) induced actin polymerization) disrupted the actin cytoskeleton in airway epithelial cells (Figure 5A), resulting in a loss of OMV fusion (Figure 5B,C), as measured by a reduction in OMV-dependent fluorescence in airway cells pretreated with cytochalasin D or wiskostatin. Thus, wiskostatin and cytochalasin blocked OMV fusion to human airway epithelial cells, demonstrating a need for N-WASP induced actin polymerization for OMV fusion to host cells.

Wiskostatin and cytochalasin D were also utilized to determine if the actin cytoskeleton is required for OMV delivery of Cif to airway epithelial cells. Cytoplasmic and endosomal fractions were purified from airway epithelial cells pretreated with vehicle, cytochalasin D or wiskostatin in the presence or absence of Cif-containing OMV. In control cells Cif localized to the endosomal fraction, as described above, whereas cytochalasin D and wiskostatin (Figure 5D) blocked the entry of Cif into the endosomal and cytoplasmic fractions. Furthermore, wiskostatin pretreatment blocked the Cif toxin-mediated reduction of CFTR from the apical membrane of airway epithelial cells (Figure 5E). Because cytochalasin D changes the rate of CFTR endocytosis, we cannot assess the effects of this inhibitor on Cif virulence factor-mediated reduction of CFTR. In addition, the purified Cif virulence factor alone did not induce morphological changes to the actin cytoskeleton (data not shown). These results reveal that Cif does not alter the cytoskeleton and establishes the requirement for an intact actin cytoskeleton, specifically N-WASP-mediated actin polymerization, for OMV fusion and virulence factor delivery to the host airway epithelial cells.

### Cif toxin is localized to the cytoplasmic face of early endosomes after entry into host cell

**Figure 6:**
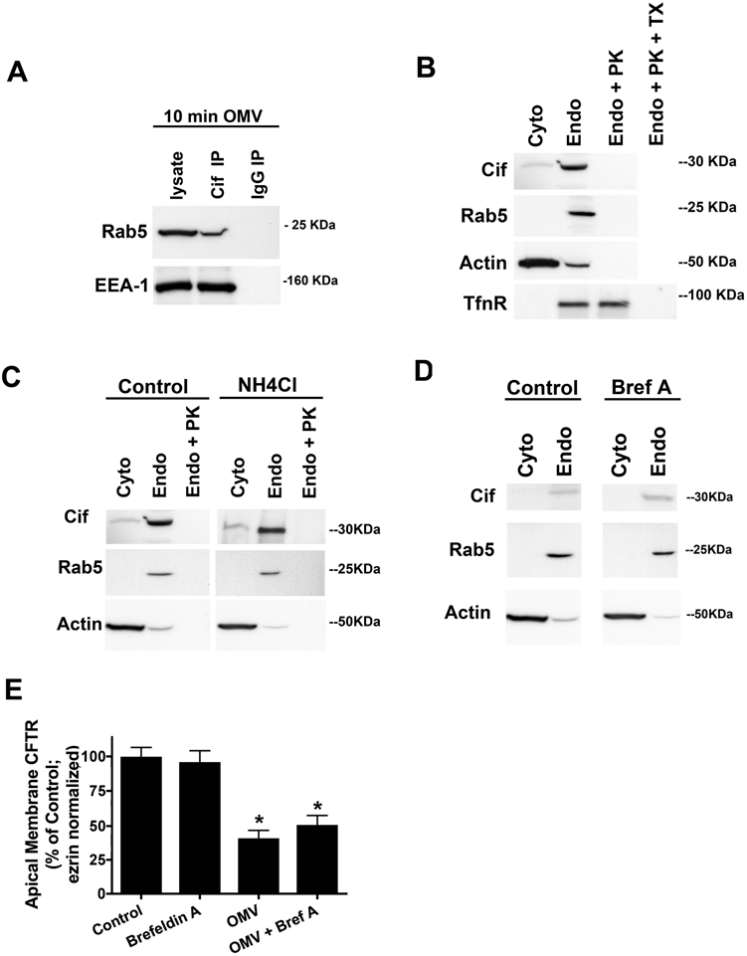
Cif virulence factor is delivered to the cytoplasm and localizes to the cytoplasmic face of early endosomes. (A) Cif localizes to the early endosomal (Rab5 GTPase, early endosomal antigen (EEA)-1 labeled) compartment after entry into airway epithelial cells. Airway epithelial cells were treated with OMV for 10 min, cells lysed, and endosomes were purified. Cif was immunoprecipitated from the endosomal fraction, and Western blot analysis was performed for Rab5 GTPase and EEA-1, early endosomal markers. IgG IP is a non-immune control immunoprecipitation experiment. (B) The ability of proteinase K (PK) to degrade Cif from the early endosomal fraction reveals that the Cif virulence factor localizes to the cytoplasmic face of early endosomes, as measured by Western blot analysis. The transferrin receptor serves as a control for a luminal endosomal protein marker, which is only exposed to proteinase K after treatment with Triton X-100 (TX). (C) Cif entry into airway epithelial cells is not altered by disruption of endosomal acidification by NH_4_Cl (5 mM). Rab5 GTPase and actin are endosomal and cytoplasmic markers, respectively. (D) Entry of Cif virulence factor into airway cells is unaffected by inhibition of retrograde transport with Brefeldin A (1 µM), as measured by Western blot analysis. Rab5 GTPase and actin are endosomal and cytoplasmic markers, respectively. (E) Retrograde transport to the endoplasmic reticulum is not required for Cif to reduce plasma membrane CFTR in airway cells. Airway epithelial cells pretreated with Brefeldin A (1 µM), which inhibits retrograde transport, were treated with OMV. Brefeldin A had no effect on the ability of Cif to reduce plasma membrane CFTR, as measured by Western blot analysis. Data are presented as mean+/−SEM. n = 3, * p<0.05 versus Control.

The data above strongly suggest that OMV deliver the Cif virulence factor into the interior of the host cell and allow this virulence factor to associate with an endosomal compartment (Figures 1A, 4D and 5D). To more precisely identify which endosomal compartment was the target of Cif, OMV were applied apically to airway cells for ten minutes, the airway cells were lysed and endosomes were purified by differential centrifugation. From the purified endosomal fraction, Cif co-immunoprecipitated with Rab5 GTPase, a marker of early endosomes, and the early endosomal antigen (EEA)-1 (Figure 6A). In contrast, Cif did not co-immunoprecipitate with Rab4 (a marker of sorting endosomes), Rab7 (a marker of late endosomes) or Rab11 (a marker of recycling endosomes) (Figure S4). Proteinase K, which does not degrade luminal endosomal proteins but can degrade proteins on the cytoplasmic face of endosomes, eliminated Cif from the endosomal fraction (Figure 6B). As expected, proteinase K did not affect the endosomal association of the transferrin receptor, a luminal endosomal protein that is resistant to proteinase K treatment [28]. However, the transferrin receptor was not resistant to proteinase K degradation in the presence of 0.1% Triton X-100, which disrupts the endosomal membrane and allows proteinase K access to luminal endosomal proteins (Figure 6B). These data reveal that OMV-delivered Cif is localized to the cytoplasmic face of the early endosomes after entry into the epithelial cell.

To determine if OMV-delivered Cif enters the host cell cytoplasm by penetrating the membrane of endosomal vesicles, cells were treated with ammonium chloride, a lysosomotropic drug that inhibits vesicle acidification, and thereby inhibits the movement of virulence factors from endosomal vesicles into the cytoplasm [3]. Ammonium chloride had no effect on the ability of Proteinase K to decrease the amount of Cif in the endosomal fractions (Figure 6C), indicating that the Cif virulence factor does not reach the cytoplasm via penetrating intracellular vesicular membranes.

Some intracellular bacteria and virulence factors move through the retrograde pathway from endosomes, to the Golgi apparatus and endoplasmic reticulum, from which they enter the host cytoplasm. However, Brefeldin A, a pharmacologic inhibitor of retrograde transport, had no effect on the entry of the Cif into the airway cell and the appearance of Cif in the endosomal fraction (Figure 6D), or the Cif-mediated reduction in apical membrane CFTR abundance (Figure 6E). Thus, our data demonstrate that OMV deliver Cif directly to the host cytoplasm rather than requiring passage across an endosomal membrane or through the retrograde transport pathway.

Interestingly, PlcH and alkaline phosphatase also localized to the endosomes after entry into the airway epithelial cells, whereas β-lactamase was detected in the cytoplasmic fraction, as determined by subcellular fractionation and Western blot analysis (data not shown). Thus, virulence factors with differing functions are distributed to different subcellular locations after entry into the host cytoplasm.

### OMVs are a physiological delivery mechanism for secreted virulence factors

**Figure 7:**
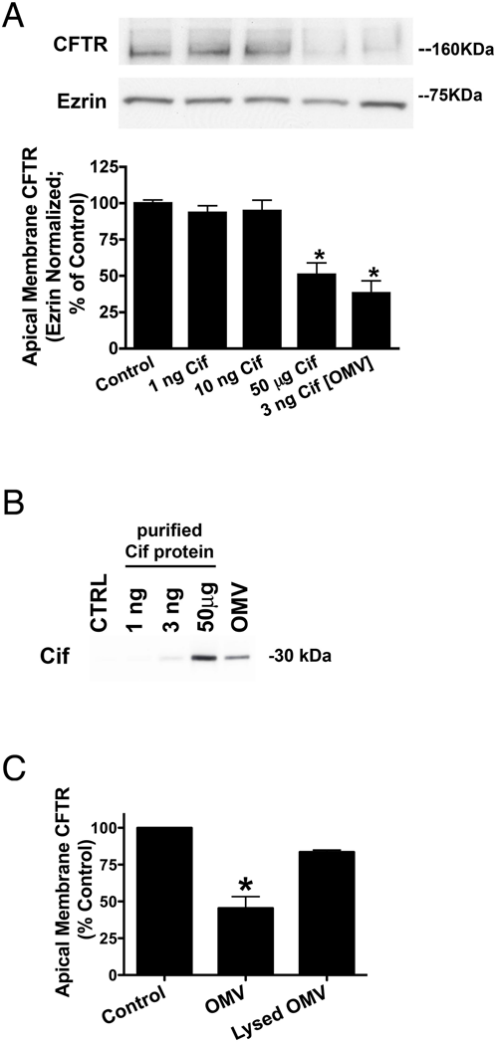
OMV required for virulence factor delivery to host airway cells. (A) OMV delivery enhances the ability of Cif to reduce plasma membrane CFTR in airway cells by 17,000-fold. Cif was applied to airway epithelial cells as 1 ng, 10 ng, 50 µg recombinant protein or 3 ng in OMV, and its effect on CFTR expression at the apical plasma membrane was measured by Western blot analysis. (B) Cif was detected in airway cells treated with 50 µg Cif or 3 ng Cif packaged in OMV. Airway epithelial cells were treated with vehicle (Control-CTRL), 1 ng, 10 ng, or 50 µg of recombinant Cif protein, or with 3 ng of Cif packaged in OMV (OMV). Cif was detected in the airway cell cytoplasm by Western blot analysis. The presence of Cif protein in the airway cell cytoplasm correlated with the ability of Cif to reduce plasma membrane CFTR (A). (C) Intact OMV required for reduction of apical membrane CFTR expression (i.e., Cif virulence factor function). Airway epithelial cells were treated with OMV or lysed OMV components, and airway epithelial cells were assayed for the Cif-mediated reduction in apical membrane CFTR, as measured by Western blot analysis. Data are presented as mean+/−SEM. n = 3, * p<0.05 versus Control.

We propose that rather than secretion of virulence factors into the surrounding medium, OMV are a physiologically- and clinically-relevant mechanism utilized by Gram-negative bacterium, in particular *P. aeruginosa*, to deliver secreted products into the host cell. In support of this hypothesis, Cif packaged in OMV was 17,000-fold more effective than purified, recombinant Cif in reducing plasma membrane CFTR, with 3 ng of Cif in OMV- reducing plasma membrane CFTR expression as effectively as 50 µg of purified, recombinant Cif protein (Figure 7A). Cif was detected in lysates of airway epithelial cells exposed to OMV (3 ng Cif) and 50 µg of recombinant Cif (Figure 7B), but Cif was not detected in cells exposed to up to 10 ng of recombinant Cif, correlating the presence of the virulence factor inside the host cell with virulence factor function. Moreover, airway epithelial cells treated with lysed OMV (Figure S2) showed a dramatic reduction in the ability of the Cif toxin to reduce apical membrane CFTR, as compared to cells treated with intact OMV (Figure 7C). Therefore, OMV-mediated delivery of virulence factors to airway epithelial cells increases the efficacy of these virulence factors in altering host cell physiology.

## Discussion

**Figure 8:**
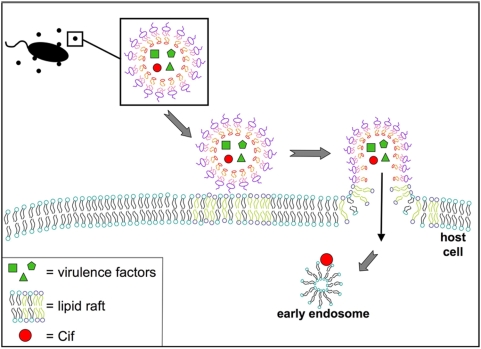
Proposed model for *P. aeruginosa* OMV fusion with airway epithelial cells. OMV are released from *P. aeruginosa*, diffuse to the host cell plasma membrane, and subsequently fuse with host cell lipid raft microdomains. Virulence factors are subsequently released into the cytoplasm of the airway epithelial cell, through an actin-dependent pathway.

We have demonstrated that *P. aeruginosa* OMV deliver multiple virulence factors, simultaneously, into host airway epithelial cells via a mechanism of OMV fusion with host cell lipid raft machinery and trafficking via an N-WASP induced actin pathway to deliver OMV cargo directly to the host cytoplasm. The OMV-delivered Cif virulence factor is then localized to the cytoplasmic face of the early endosomal compartment (Figure 8). *E. coli* OMV association with host cells had previously been shown to be sensitive to Filipin III treatment, and thus was proposed to be lipid raft-dependent, but whether the OMV actually delivered cargo into the host cells and the mechanism by which this occurred was not characterized [15]. Fiocca *et al.* demonstrated that VacA packaged in OMV from *H. pylori* was internalized into a cytoplasmic vacuole in gastric epithelial cells, but did not investigate a mechanism [29]. We propose the first mechanism for the entry and intracellular fate of OMV-delivered bacterial virulence factors.

Considering the pioneering work of Beveridge to characterize OMV and our current mechanistic studies, we propose that OMV-mediated virulence factor delivery should be considered for designation as a secretion system [4]–[7]. Like the T3SS, OMV can deliver bacterial proteins directly to the host cell cytoplasm without releasing the naked bacterial proteins into the extracellular environment where they could be degraded by secreted proteases [30]–[32]. OMV deliver fully-folded, enzymatically-active secreted virulence factors into host cells, ready for immediate action upon delivery. By delivering multiple, active OMV-packaged virulence factors, the pathogen may be able to impact the host on multiple levels. For example, simultaneously altering epithelial cell function by perturbing surfactant abundance or tight junction integrity, and the innate immune response to bacteria by stimulating pro-inflammatory cytokine production [14], [33]–[36]. Based on our studies in *P. aeruginosa* and published reports of OMV production by *E. coli*, *H. pylori*, *A. actinomycetemcomitans*, *V. cholerae* and *N. meningitidis*, it is likely that other bacteria package multiple secreted virulence factors in OMV for efficient transfer to host cells and thus, the studies proposed here likely represent a general strategy utilized by Gram-negative bacteria in their interactions with the host [10]–[12],[37],[38].

In contrast to known secretion systems, OMV-mediated direct delivery of bacterial proteins to the host can occur at a distance, and in the absence of bacteria, thus obviating the need for the pathogen to interact directly with the host cell to cause cellular cytotoxicity and alter host cell biology to promote colonization. Furthermore, OMV can deliver bacterial factors across host barriers, such as mucus layers. We believe that our work should prompt those studying bacterial pathogens to reconsider how secreted virulence factors impact host cells. That is, our data suggest that secreted virulence factors are not released individually into the surrounding milieu where they may randomly contact the surface of the host cell, but are released in a strategic manner, packaged with multiple virulence factors in OMV for coordinated delivery directly into the host cell cytoplasm. It is also possible that OMV provide a mechanism for delivering a concentrated bolus of virulence factors to the host, instead of individual toxins being delivered one at a time to the host cell. Moreover, OMV-mediated, long distance delivery of virulence factors might help explain observations such as, bacterial colonization of catheters causing systemic symptoms in kidney dialysis patients, ocular keratitis occurring in patients who do not have cultivatable pathogens, and the significant lung damage in cystic fibrosis, bronchiectasis, and chronic obstructrive pulmonary disease patients resulting from chronic infections with *P. aeruginosa* suspended in mucus above the airway epithelium. This mechanism of OMV-mediated protein secretion is reminiscent of the long distance delivery of signaling proteins between and among eukaryotic cells via exosomes [39], and may represent a general protein secretion strategy used by both pathogen and host.

## Materials and Methods

### Antibodies and reagents

The antibodies used were: rabbit anti-Cif antibody (Covance Research Products, Denver, Pa [16]); rabbit anti-OprF antibody (a generous gift from Nobuhiko Nomura, Graduate School of Life and Environmental Sciences, University of Tsukuba); rabbit anti-pilus antibody (a generous gift from Michael Zegans, Dartmouth Medical School); goat anti-phospholipase C-H antibody (a generous gift from Michael Vasil); mouse anti-human CFTR C-terminus antibody (clone 24-1; R&D systems, Minneapolis, MN); mouse anti-CFTR antibody (clone M3A7; Upstate Biotechnology, Lake Placid, NY); mouse anti-EEA1 antibody, mouse anti-ezrin antibody, mouse anti-flotillin-1 antibody, mouse anti-Rab5 antibody, mouse anti-actin antibody (BD Biosciences, San Jose, CA); cholera toxin B subunit-FITC (Sigma-Aldrich, St. Louis, MO); rabbit anti-GPIp137 antibody (Abgent, San Diego, CA); Alexa 647-conjugated phalloidin (Molecular Probes, Carlsbad, CA); rabbit anti-Rab4 antibody, rabbit anti-Rab7 antibody (Santa Cruz Biotechnology, Santa Cruz, CA); mouse anti-Rab11 antibody, mouse anti-transferrin receptor antibody (Zymed, San Francisco, CA); mouse anti-β lactamase antibody (Novus Biologicals, Littleton, CO); rabbit anti-alkaline phosphatase antibody (GeneTex, Inc., San Antonio, TX) and horseradish peroxidase-conjugated goat anti-mouse and goat anti-rabbit secondary antibodies (Bio-Rad, Hercules, CA). Other reagents include: Filipin III complex, ammonium chloride, Optiprep, proteinase K, and cytochalasin D (Sigma-Aldrich), wiskostatin (Calbiochem, San Diego, CA), Triton X-100 (Bio-Rad, Hercules, CA). All antibodies and reagents were used at the concentrations recommended by the manufacturers or as indicated in the figure legends.

### Cell culture

Two airway epithelial cell lines were studied to examine outer membrane vesicle fusion and toxin delivery to host epithelial cells. First, human bronchial epithelial CFBE cells (ΔF508/ΔF508) were stably transduced with WT-CFTR (generous gift from Dr. J. P. Clancy, University of Alabama at Birmingham, Birmingham, AL; hereafter referred to as airway epithelial cells) [40]. CFBE WT-CFTR cells were polarized on 24-mm transwell permeable supports (0.4-µm-pore size; Corning, Corning, NY) coated with vitrogen plating medium containing human fibronectin, as described previously [41]. Second, human airway epithelial cells (Calu-3) were obtained from the American Type Culture Collection (Manassas, VA) and polarized on 24-mm transwell permeable supports, as described previously [42].

### *Pseudomonas aeruginosa* cultures

Lysogeny broth (LB) was inoculated with *P. aeruginosa* strain UCBPP-PA14 (PA14) [43] and cultures were prepared as previously reported [16].

### Outer membrane vesicle purification

OMV were purified using a differential centrifugation and discontinuous Optiprep gradient protocol adapted from Bauman *et al.* [14] OMV were lysed, when noted, with 100 mM EDTA at 37°C for 60 minutes.

### Cell compartment fractionation

To study the localization of the Cif toxin after OMV fusion with the airway epithelial cell, differential centrifugation and fractionation techniques were used to isolate cytosolic and early endosomal compartments. Early endosomes were isolated using a protocol adapted from Butterworth *et al.* [44].

### Immunoprecipitation

To characterize proteins interacting with the Cif toxin in lipid raft microdomains, Cif was immunoprecipitated from airway epithelial cell lipid raft fractions by methods described previously [45].

### Detergent-resistant membrane fractionation

To determine if OMV fuse with lipid raft microdomains of the host, detergent-resistant membranes were purified from airway epithelial cells that had been exposed to OMV. These studies were performed using a discontinuous Optiprep gradient in a protocol adapted from Pike *et al.* [46].

### OMV fusion assay

To monitor the fusion of OMV with airway epithelial cells, OMV were fluorescently labeled with a probe that fluoresces upon membrane fusion. OMV purified with the method described above were resuspended in labeling buffer (50 mM Na_2_CO_3_, 100 mM NaCl, pH 9.2). Rhodamine isothiocyanate B-R18 (Molecular Probes), which integrates in the membrane of the OMV, was added at a concentration of 1 mg/ml for 1 hour at 25°C, followed by ultracentrifugation at 52,000×g for 30 min at 4°C. Rhodamine isothiocyanate B-R18 fluorescence is quenched at high concentrations in bilayer membranes, and fluorescence is dequenched when the probe is diluted upon vesicle fusion. Subsequently, rhodamine labeled-OMV were resuspended in PBS (0.2 M NaCl) and pelleted at 52,000×g for 30 min a 4°C. After a final centrifugation step, the labeled-OMV were resuspended in 1 ml PBS (0.2 M NaCl) containing a protease inhibitor cocktail tablet (Complete Protease Inhibitor Tablet, Roche). Labeled-OMV were applied to the apical side of airway epithelial cells at 1∶4 dilution of labeled-OMV to Earle's Minimal Medium (MEM, Invitrogen) and fluorescence was detected over time as indicated on a fluorescent plate reader (Ex 570 nm; Em 595 nm). Fluorescence intensity was normalized for fluorescence detected by labeled-OMV in the absence of airway epithelial cells at the indicated time points.

### Confocal microscopy

To visualize the fusion and localization of OMV with airway epithelial cells, rhodamine R18-labeled OMV (see OMV Fusion Assay method) were applied to the apical membrane of cells and confocal sections were captured over time. Airway epithelial cells were seeded at 0.1×10^6^ on collagen-coated, glass-bottom MatTek dishes (MatTek, Ashland, MA) and grown for 6–7 days in culture at 37°C. For wheat germ agglutin (WGA, which labels the plasma membrane) studies, nonpermeabilized cells were incubated for 5 minutes with Alexa-647 WGA (1 µg/ml, 37°C; Molecular Probes) following 15-minute vesicle incubation. Z-stacks of all labeled cells were acquired with a Nikon Sweptfield confocal microscope (Apo TIRF 60× oil immersion 1.49 NA objective) fitted with a QuantEM:512sc camera (Photometrics, Tuscon, AZ) and Elements 2.2 software (Nikon, Inc.). For OMV fusion experiments, a single confocal section (0.4 µm) at the apical membrane of the airway epithelial cells is presented. Experiments were repeated three times, with five fields imaged for each experiment.

### Cell-surface biotinylations and Western blot analysis

To examine the effect of OMV on the apical membrane expression of CFTR, cell surface biotinylation was performed as described in detail previously by our laboratory [41]. Protein band intensity was analyzed as described previously using NIH image software, version 1.63 (Wayne Rasband, NIH, USA; http://rsb.info.nih.gov).

### Cytotoxicity assay

To determine if *P. aeruginosa* OMV are cytotoxic to airway cells, cells were incubated with OMV in serum-free media for the indicated time points. Cytotoxicity was measured using the CellTiter 96 AQ_ueous_ One Solution Reagent (Promega, Madison, WI), according to the manufacturer's protocol.

### Data analysis and statistics

Statistical analysis of the data was performed using Graphpad Prism version 4.0 for Macintosh (Graphpad, San Diego, CA). Means were compared using a Students t-test or one-way ANOVA, followed by a Tukey-Kramer post hoc test using a 95% confidence interval. Data are expressed as means+/−SEM.

### Accession numbers

Cif (PA2934, NP 251624.1); PlcH (PA0844, YP 792433.1); alkaline phosphatase (PA3296, YP 789857.1); β-lactamase (PA1797, YP 791446.1); N-WASP (NP 003932); Omp85 (PA3648, YP 789516); GPIp137 (NP 005889).

## Supporting Information

Figure S1 The Cif virulence factor is packaged in *P. aeruginosa* OMV. From an overnight *P. aeruginosa* PA14 culture, Optiprep density gradient centrifugation was utilized to purify OMV from the bacteria and possible contaminants, including pilus (PilA). Purified OMVs retrieved from fractions 2 and 3 were pooled for use in all experiments described. Experiment repeated three times; representative blot shown.
(0.35 MB TIF)

Figure S2 EDTA effectively lyses OMV. EDTA (0.1 M) disrupted OMV membranes to allow proteinase K (PK)-mediated degradation of Cif, an intravesicular OMV component, as measured by Western blot analysis. Experiment repeated three times; representative blot shown.
(0.18 MB TIF)

Figure S3 Cif virulence factor is an intravesicular OMV component. Isolated OMV treated with Proteinase K (PK: 100 µg/ml) for 1 h at 37°C to degrade proteins on the exterior of OMV. Δ*cif*: OMV purified from a *P. aeruginosa* Δcif mutant strain. Experiment repeated three times; representative blot shown.
(0.35 MB TIF)

Figure S4 Cif does not localize to Rab4, Rab7, or Rab11-labeled endosomes. Cif does not localize to the sorting endosomal (Rab4 GTPase-labeled), late endosomal (Rab7 GTPase-labeled), or recycling endosomal (Rab11 GTPase-labeled) compartments after entry into airway epithelial cells. Airway epithelial cells were treated with OMV for 10 min, cells lysed, and endosomes were purified. Cif was immunoprecipitated from the endosomal fraction and Western blot analysis was performed for Rab4, 7, and 11 GTPases. IgG IP is a non-immune control immunoprecipitation experiment.
(0.60 MB TIF)
