# Supplementary figures and images for: Long-Distance Delivery of Bacterial Virulence Factors by Pseudomonas aeruginosa Outer Membrane Vesicles
Source: PLoS Pathog. 2009 Apr 10;5(4):e1000382. doi: 10.1371/journal.ppat.1000382 (PMC2661024; doi:10.1371/journal.ppat.1000382)

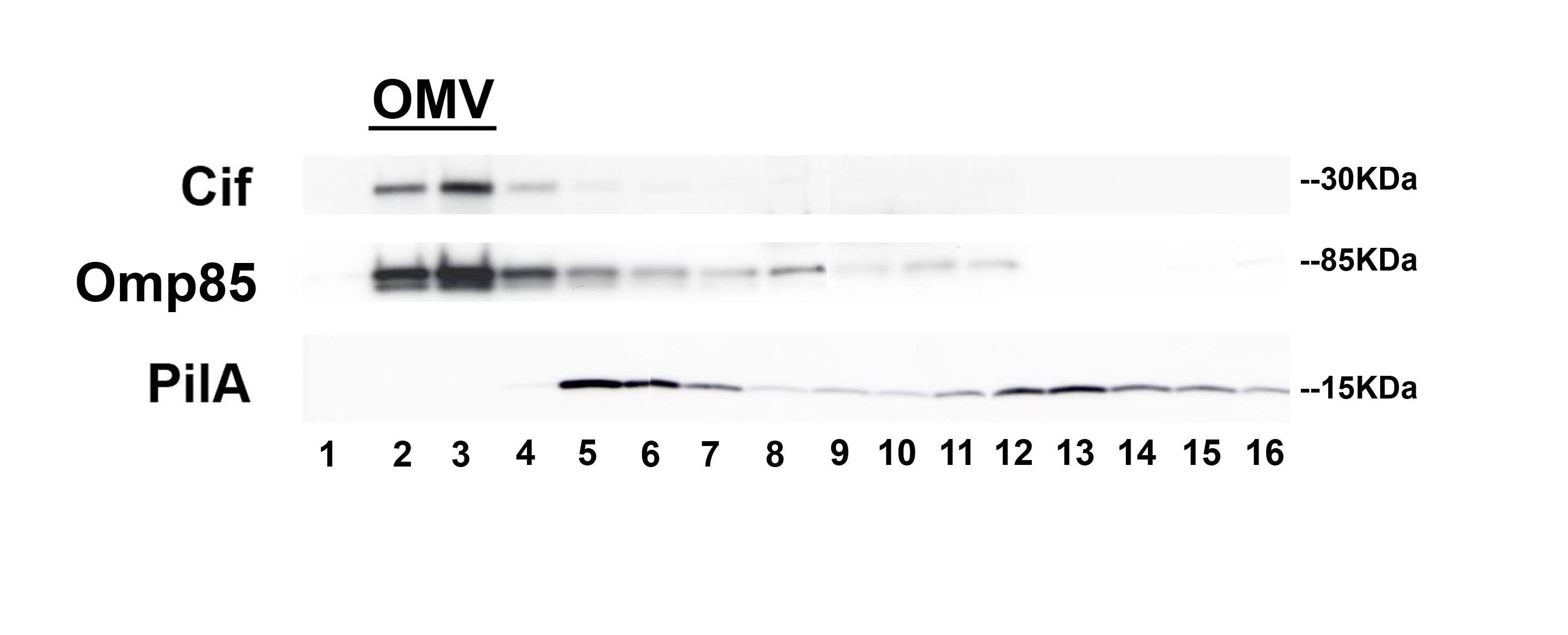

Supplement: Figure S1 — The Cif virulence factor is packaged in P. aeruginosa OMV. From an overnight P. aeruginosa PA14 culture, Optiprep density gradient centrifugation was utilized to purify OMV from the bacteria and possible contaminants, including pilus (PilA). Purified OMVs retrieved from fractions 2 and 3 were pooled for use in all experiments described. Experiment repeated three times; representative blot shown. (0.35 MB TIF) [file ppat.1000382.s001.tif]

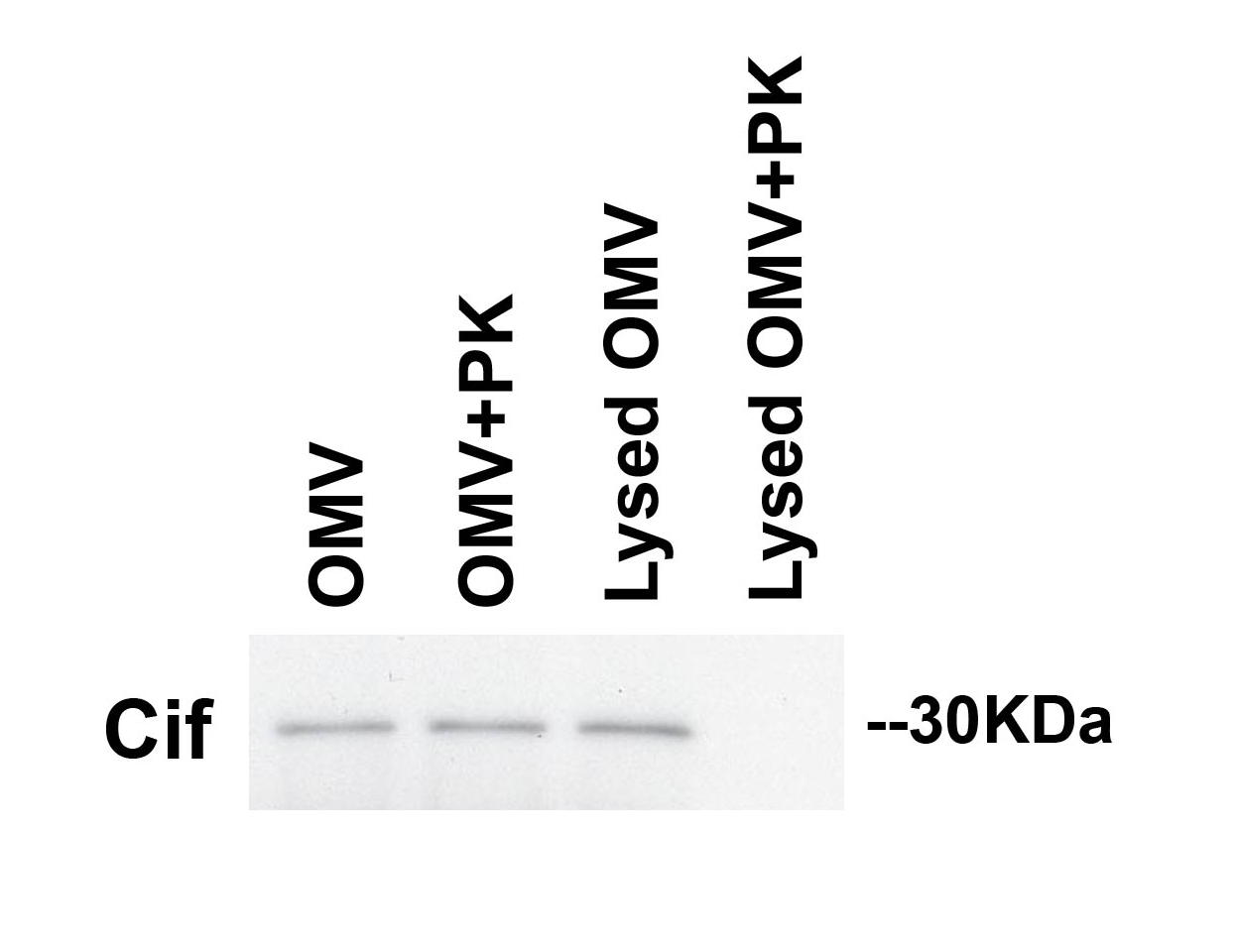

Supplement: Figure S2 — EDTA effectively lyses OMV. EDTA (0.1 M) disrupted OMV membranes to allow proteinase K (PK)-mediated degradation of Cif, an intravesicular OMV component, as measured by Western blot analysis. Experiment repeated three times; representative blot shown. (0.18 MB TIF) [file ppat.1000382.s002.tif]

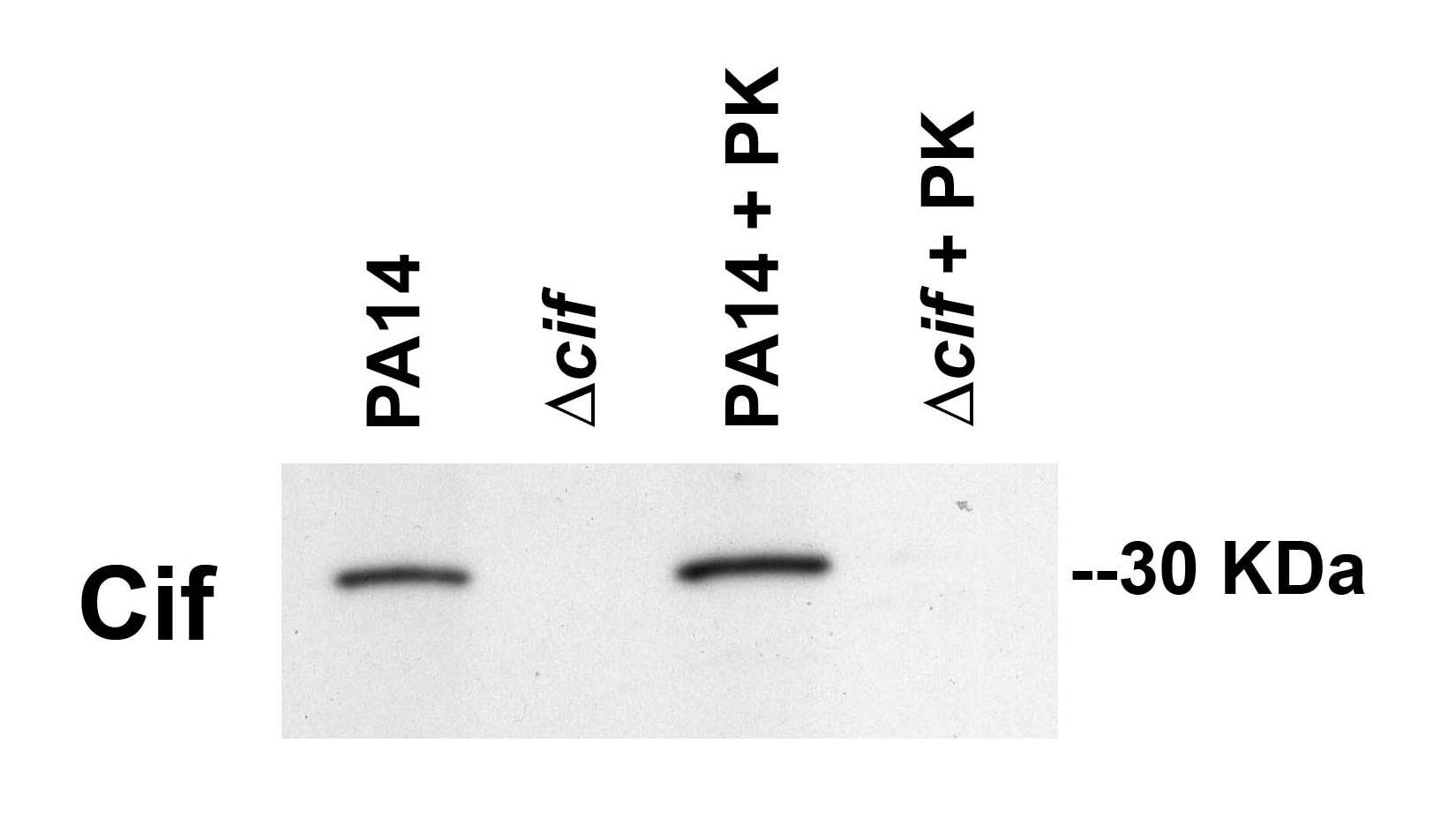

Supplement: Figure S3 — Cif virulence factor is an intravesicular OMV component. Isolated OMV treated with Proteinase K (PK: 100 µg/ml) for 1 h at 37°C to degrade proteins on the exterior of OMV. Δcif: OMV purified from a P. aeruginosa Δcif mutant strain. Experiment repeated three times; representative blot shown. (0.35 MB TIF) [file ppat.1000382.s003.tif]

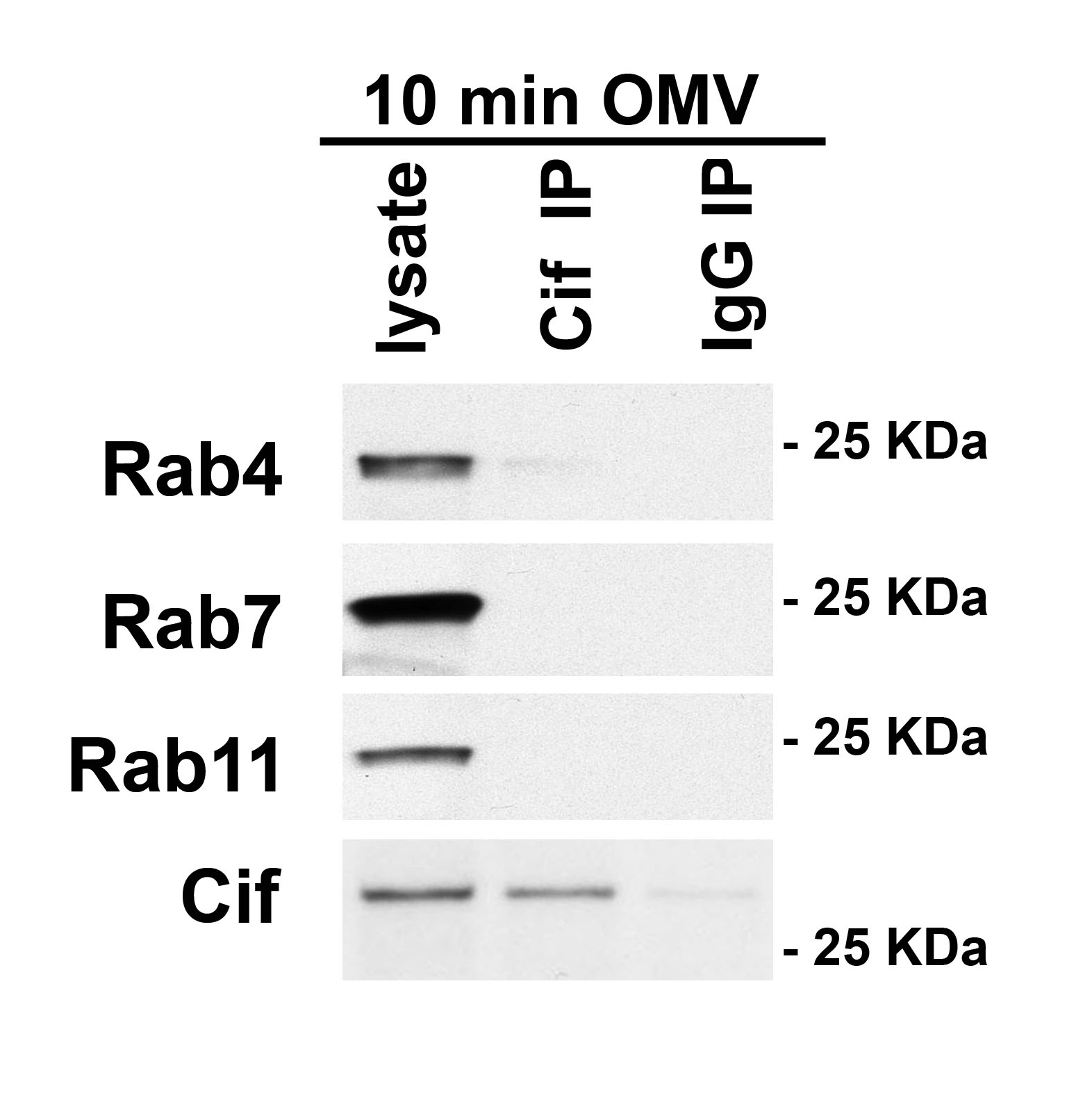

Supplement: Figure S4 — Cif does not localize to Rab4, Rab7, or Rab11-labeled endosomes. Cif does not localize to the sorting endosomal (Rab4 GTPase-labeled), late endosomal (Rab7 GTPase-labeled), or recycling endosomal (Rab11 GTPase-labeled) compartments after entry into airway epithelial cells. Airway epithelial cells were treated with OMV for 10 min, cells lysed, and endosomes were purified. Cif was immunoprecipitated from the endosomal fraction and Western blot analysis was performed for Rab4, 7, and 11 GTPases. IgG IP is a non-immune control immunoprecipitation experiment. (0.60 MB TIF) [file ppat.1000382.s004.tif]
